# Detection of disease‐causing mutations in prostate cancer by NGS sequencing

**DOI:** 10.1002/cbin.11803

**Published:** 2022-04-06

**Authors:** Alessandra Mangolini, Christian Rocca, Cristian Bassi, Carmelo Ippolito, Massimo Negrini, Lucio Dell'Atti, Giovanni Lanza, Roberta Gafà, Nicoletta Bianchi, Paolo Pinton, Gianluca Aguiari

**Affiliations:** ^1^ Department of Neuroscience and Rehabilitation University of Ferrara Ferrara Italy; ^2^ UO Urology, St Anna Hospital Ferrara Italy; ^3^ Department of Translational Medicine University of Ferrara Ferrara Italy; ^4^ Division of Urology, Department of Clinical, Special and Dental Science, University Hospital “Ospedali Riuniti” Marche Polytechnic University Ancona Italy; ^5^ Department of Medical Sciences University of Ferrara Ferrara Italy

**Keywords:** gene mutations, hereditary cancer‐predisposing syndrome, NGS, prognosis, prostate cancer, signaling pathways

## Abstract

Gene mutations may affect the fate of many tumors including prostate cancer (PCa); therefore, the research of specific mutations associated with tumor outcomes might help the urologist to identify the best therapy for PCa patients such as surgical resection, adjuvant therapy or active surveillance. Genomic DNA (gDNA) was extracted from 48 paraffin‐embedded PCa samples and normal paired tissues. Next, gDNA was amplified and analyzed by next‐generation sequencing (NGS) using a specific gene panel for PCa. Raw data were refined to exclude false‐positive mutations; thus, variants with coverage and frequency lower than 100× and 5%, respectively were removed. Mutation significance was processed by Genomic Evolutionary Rate Profiling, ClinVar, and Varsome tools. Most of 3000 mutations (80%) were single nucleotide variants and the remaining 20% indels. After raw data elaboration, 312 variants were selected. Most mutated genes were *KMT2D* (26.45%), *FOXA1* (16.13%), *ATM* (15.81%), *ZFHX3* (9.35%), *TP53* (8.06%), and *APC* (5.48%). Hot spot mutations in *FOXA1, ATM*, *ZFHX3*, *SPOP*, and *MED12* were also found. Truncating mutations of *ATM*, lesions lying in hot spot regions of *SPOP* and *FOXA1* as well as mutations of *TP53* correlated with poor prognosis. Importantly, we have also found some germline mutations associated with hereditary cancer‐predisposing syndrome. gDNA sequencing of 48 cancer tissues by NGS allowed to detect new tumor variants as well as confirmed lesions in genes linked to prostate cancer. Overall, somatic and germline mutations linked to good/poor prognosis could represent new prognostic tools to improve the management of PCa patients.

## 1 INTRODUCTION

Prostate cancer (PCa) is the most common noncutaneous cancer of man in Europe, where the highest incidence of clinically diagnosed PCa in Northern and Western part of Europe was found (Mottet et al., 2021). In absence of early diagnosis, the mortality rate for PCa patients is very high representing about the sixth most fatal cancer in man (Dejous & Krishnan, 2020). Patients with high‐grade disease characterized by T3‐4 stage, lymph node invasion, or an extraprostatic extension have a high‐risk (most of 40%) of disease recurrence after 5–10 years from the diagnosis (Spratt et al., 2018). Currently, the main tool for PCa detection is the analysis of prostate‐specific antigen (PSA) serum levels combined with direct rectal examination (DRE). However, PSA serum detection remains one of the most controversial topics in the urologic literature, since it leads to overdiagnosis and overtreatment of positive subjects (Mottet et al., 2021). Moreover, neither overall survival (OS) nor cancer‐specific survival (CSS) benefits in patients screened by PSA were observed (Mottet et al., 2021). Prostate cancer treatments are dependent on the staging of tumor and includes active surveillance (AS), surgery, hormone therapy, radiotherapy, or a combination of these treatments (Dejous & Krishnan, 2020). Moreover, early diagnosis and disease outcome prediction are crucial points to increase patient OS (Dejous & Krishnan, 2020). Genomic alterations deeply affected cancer biology and disease course in tumors including PCa. In particular, the fusion of the genes ERG and TMPRSS2 is one of most frequent genomic alterations observed in PCa (Gasi Tandefelt et al., 2014). Moreover, somatic gene mutations linked to tumor progression such as oncogenes or tumor suppressor genes were also identified (Gandhi et al., 2018). The detection of gene mutations linked to PCa outcome might improve the knowledge of this tumor increasing prognostic tools and therapeutic options.

## 2 MATERIALS AND METHODS

### 2.1 Materials

Disposable RNAse/DNAse free plastic material was purchased by EuroClone. Ion AmpliSeq™ Custom and Community Panels, Ion AmpliSeq™ Library Kits 2.0, Ion Xpress™ Barcode Adapters 1‐96 Kits, Ion PGM™ Hi‐Q™ View OT2 Kit, Ion Sphere Quality Control Kit, Ion PGM™ Hi‑Q™ View Sequencing Kit, Ion 316™ Chip Kit v2 BC, and Qubit® dsDNA HS Assay Kit were obtained from Thermo Fisher Scientifics. Agencourt® AMPure® XP Kit was purchased from Beckman Coulter; QIAmp FFPE tissues kit was obtained from Qiagen.

### 2.2 Tissue collection

Paraffin‐embedded tumor samples (23 GS6, 11 GS7, 11 GS8, and 3 GS9) from 48 patients underwent to radical prostatectomy in the years 2010–2015 were collected. The diagnosis of cancer samples was evaluated by genitourinary pathologist on hematoxiline and eosine (H&E)‐stained slides. Selected samples (both tumor and normal tissues from the same patient) were cut into 8 × 10 µm sections with the last H&E stained 4 µm sections to confirm tumor cellularity. This is a retrospective study approved by Ethics Committee (no 151095). A written consent regarding tissue analysis and outcome data for all cases enrolled was collected. This study follows the guidelines of Helsinki Declaration.

### 2.3 Prostate panel design

A prostate cancer‐specific Ion AmpliSeq™ Custom and Community Panel (PC panel) was designed through the AmpliSeq.com program by selecting target regions of 16 genes (*APC, AR, ATM, CDK12, CHD1, COL5A1, FOXA1, MED12, KMT2D, OR5L1, PIK3CA, PTEN, RB1, SPOP, TP53*, and *ZFHX3*) that are the more frequently mutated in prostate tumor (Frank et al., 2018; Robinson et al., 2015). The PC panel consists of two DNA primer pools (pool 1: 337 amplicons, pool 2: 331 amplicons) capable to amplified coding regions of maximum 150 bp in length to ensure optimal amplification. All gene information of PC panel was inserted in Table 1.

**Table 1:** Genes related to prostate cancer.

Gene	Name	Chromosome	Exon coverage	Protein	Function
PIK3CA	Phosphatidylinositol‐4,5‐bisphosphate 3‐kinase catalytic subunit alpha	3	2,5,8,10,21	PI3K subunit	Cell proliferation migration and survival
APC	Adenomatosis Polyposis Coli	5	6,11	WNT signaling pathway regulator	Tumor Suppressor, cell migration, adhesion, apoptosis
CHD1	Chromodomain helicase DNA binding protein 1	5	3,12,13,14,18,29,34,35	ATP‐dependent chromatin‐remodeling factor	Negative regulator of DNA replication
COL5A1	Collagen, type V, alpha 1	9	3,7,24,33,46	A component of type V collagen	Cellular component organization, cell adhesion
PTEN	Phosphatase and tensin homolog	10	2,3,4,5,6,7,8	Protein Phosphatase	Tumor suppressor, cell division regulator
OR5L1	Olfactory receptor family 5 subfamily L member 1	11	All coding sequence	G‐protein‐coupled receptor	Sensory transduction
ATM	Ataxia telangiectasia mutated	11	All coding sequence	Serine/threonine kinase	DNA repair, cell cycle control
KMT2D	Lysine methyltransferase 2D	12	All coding sequence	Histone methyltransferase	Tumor suppressor
RB1	RB transcriptional corepressor 1	13	3,7,12,19,23	transcription repressor	Tumor suppressor
FOXA1	Forkhead box protein A1	14	2	DNA‐binding protein	Cofactor for steroid receptor binding
ZFHX3	Zinc finger homeobox 3	16	2,8,9,10	Transcription factor	Tumor suppressor
TP53	Tumor protein p53	17	2,4,5,6,7,8,9,10	DNA repair regulator	Tumor suppressor
CDK12	Cyclin dependent kinase 12	17	All coding sequence	Cyclin‐dependent kinase	Transcription elongation, DNA repair, and genomic stability regulator
SPOP	Speckle type BTB/POZ protein	17	5,6,11	transcription regulator	Gene transcription modulator
AR	Androgen receptor	X	1,4,5,8	Hormone receptor	Androgen‐responsive gene regulator
MED12	Mediator complex subunit 12	X	4,9,15,26,28,31	Transcription factor binding	Mediator complex for RNA Polymerase II transcription machinery

*Note*: Gene acronym, location, coverage, and function are indicated.

### 2.4 Genomic DNA extraction, sample enrichment, and NGS sequencing

Genomic DNA (gDNA) was extracted with QIAmp FFPE tissues kit (Qiagen) according to the manufacturer's instructions. gDNA quantity and quality were assessed using the Qubit ® 2.0 photometer (Thermo Fisher Scientific) and the Qubit ® dsDNA HS Assay Kit. gDNA was diluted at the final concentration of 5 ng/μl with deionized water. Libraries were prepared from 10 ng of gDNA using the PC Panel. Overall, gDNA was subjected to library preparation according with Ion Ampliseq Libreries kit 2.0 (Thermo Fisher Scientific). Target regions were initially amplified (20 PCR cycles) with a multiple PCR; after thermal cycling amplification, amplicons produced from pool 1 and pool 2 were combined and partially digested. Next, they were subjected to ligation of barcoded adapters and purified. Before sequencing, libraries were quantified using the Agilent™ 2100 Bioanalyzer™ (Agilent Genomics) and dilute to 100 pM. Barcoded libraries, combined for maximizing chip use, labor, and costs, were clonally amplified by emulsion PCR using OneTouch™ Instrument (Thermo Fisher Scientific) and enriched by the OneTouch™ ES Instrument (Thermo Fisher Scientific) using the Ion PGM™ Hi‐Q™ View OT2 Kit, following the manufacturer's instructions. Library quality control was performed using the Ion Sphere Quality Control Kit according to the manufacturer's instructions, ensuring that 10%–30% of template positive Ion Sphere particles (ISP) were targeted in the emPCR reaction. Finally, sequencing was performed on the Ion PGM™ (Thermo Fisher Scientific) with the Ion PGM™ Hi‐Q View™ Sequencing Kit (Thermo Fisher Scientific), loading barcoded samples (8 samples) into a 316 v.2 BD chip (Rothberg et al., 2011).

### 2.5 Data elaboration

Sequencing data analysis was conducted by using Torrent Suite software v. 5.0 (Thermo Fisher Scientific). The alignment against a reference genome (hg19) was performed by using the Torrent Mapping Alignment Program after low‐quality reads removal and adapter sequences trimming. The Torrent Variant Caller plugin was used to identify variations from the reference sequence. To identify pathogenic variations, mutations that did not affect the protein‐coding regions (intronic, 3′ and 5′ untranslated region [UTR] variations, and silent exonic mutations) were filtered out. All detected variants were manually reviewed with the Integrative Genomics Viewer (IGV V.2.1, Broad Institute). Genomic Evolutionary Rate Profiling (GERP) tool was used to predict the effect of missense mutations on the protein and calculate their conservation scores (Deshpande et al., 2018). This analysis was improved by using ClinVar and Varsome databases. For high confidence detection of somatic mutations present in heterogeneous cancer tissues, samples with coverage less than 100× and mutation frequency lower than 5% were excluded.

## 3 RESULTS

### 3.1 Detection of gene mutations by NGS analysis

**Figure 1:**
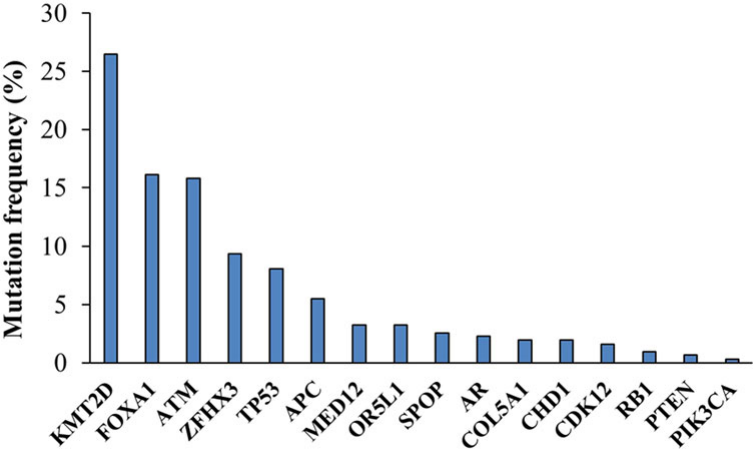
Mutation frequency of genes related to prostate cancer detected in a cohort of 48 subjects by NGS analysis. The most mutated genes are *KMT2D*, *FOXA1*, and *ATM*, while in *RB1*, *PTEN*, and *PIK3CA* few variants were detected. NGS, next‐generation sequencing

Genomic sequences of 48 PCa tissues and their paired normal samples were subjected to NGS analysis for identifying disease‐causing mutations. After row data processing and the exclusion of synonymous variants, 312 mutations (5 small deletions, 1 duplication, and 306 SNVs) widespread along the exonic sequences of 16 genes related to prostate carcinoma were detected (Table S1). Three deletions were in frame, while the other two led to transcript frameshift as well as the only duplication observed in our cohort. Among the 306 SNVs, three were stop codon while the remaining 303 were missense mutations. Overall, we found 77 germline and 235 somatic mutations. Sixty‐six germline mutations were considered polymorphic variants, while the other 11 were considered possible hereditary‐causing cancer lesions. Regarding the 235 somatic mutations, 67 were classified as benign, 28 as uncertain significance, and 140 as likely pathogenic (Table S1). As shown in Figure 1, the percentage distribution of all mutations detected in genes of the PC panel was the following: *KMT2D* (26.45), *FOXA1* (16.13), *ATM* (15.81), *ZFHX3* (9.35), *TP53* (8.06), *APC* (5.48), *MED12* (3.23), *OR5L1* (3.23), *SPOP* (2.58), *AR* (2.26), *COL5A1* (1.94), *CHD1* (1.94), *CDK12* (1.61), *RB1* (0.97), *PTEN* (0.65), and *PIK3CA* (0.32).

### 3.2 Recurrent mutations

**Figure 2:**
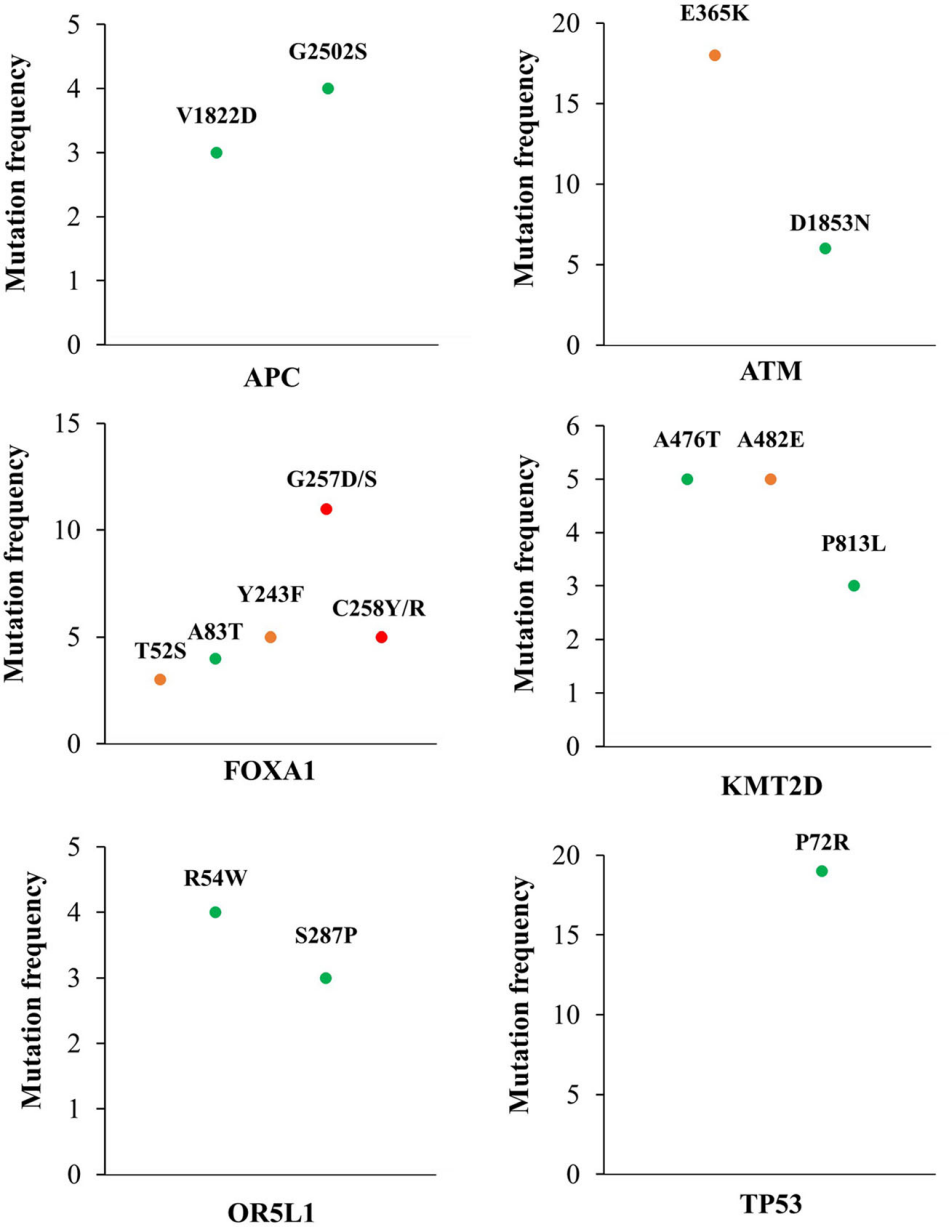
Recurrent mutations found in patients with prostate cancer. Genes with recurrent pathogenic mutations are *ATM* and *FOXA1*, while variants found in *APC*, *OR5L1*, *KMT2D*, and *TP53* are likely benign except for the A482E substitution in *KMT2D*, which is considered as uncertain significance. Red dots = likely pathogenic; orange = uncertain significance; green = likely benign.

We identified some recurrent mutations in different subjects (Figure 2). In particular, the V1822D (*n* = 3) and G2502S (*n* = 4) substitutions in *APC* were considered benign variants.

The mutation E365K (*n* = 18) in *ATM* was processed as uncertain significance and showed a high frequency in our cohort (37.5%). In this gene the benign variant D1853N (*n* = 6) was also identified. Recurrent mutations were also detected in *FOXA1*; the variants Y243F (*n* = 5) and T52S (*n* = 3) were considered as uncertain significance, while A83T (*n* = 4) was processed as benign. Conversely, the variants G257D/S (*n* = 11) and C258Y/R (*n* = 5) were supposedly pathological mutations.

In *KMT2D*, the variants A476T (*n* = 5) and P813L (*n* = 3) were benign while the mutation A482E (*n* = 5) was considered as uncertain significance. Finally, the benign variants R54W (*n* = 4) and S287P (*n* = 3) in *OR5L1* as well as P72R in *TP53* (*n* = 19) were also identified. Interestingly, the mutation P72R was the germline variant most frequent our cohort, which is present in approximately 40% of cases.

### 3.3 Hotspot mutations

**Figure 3:**
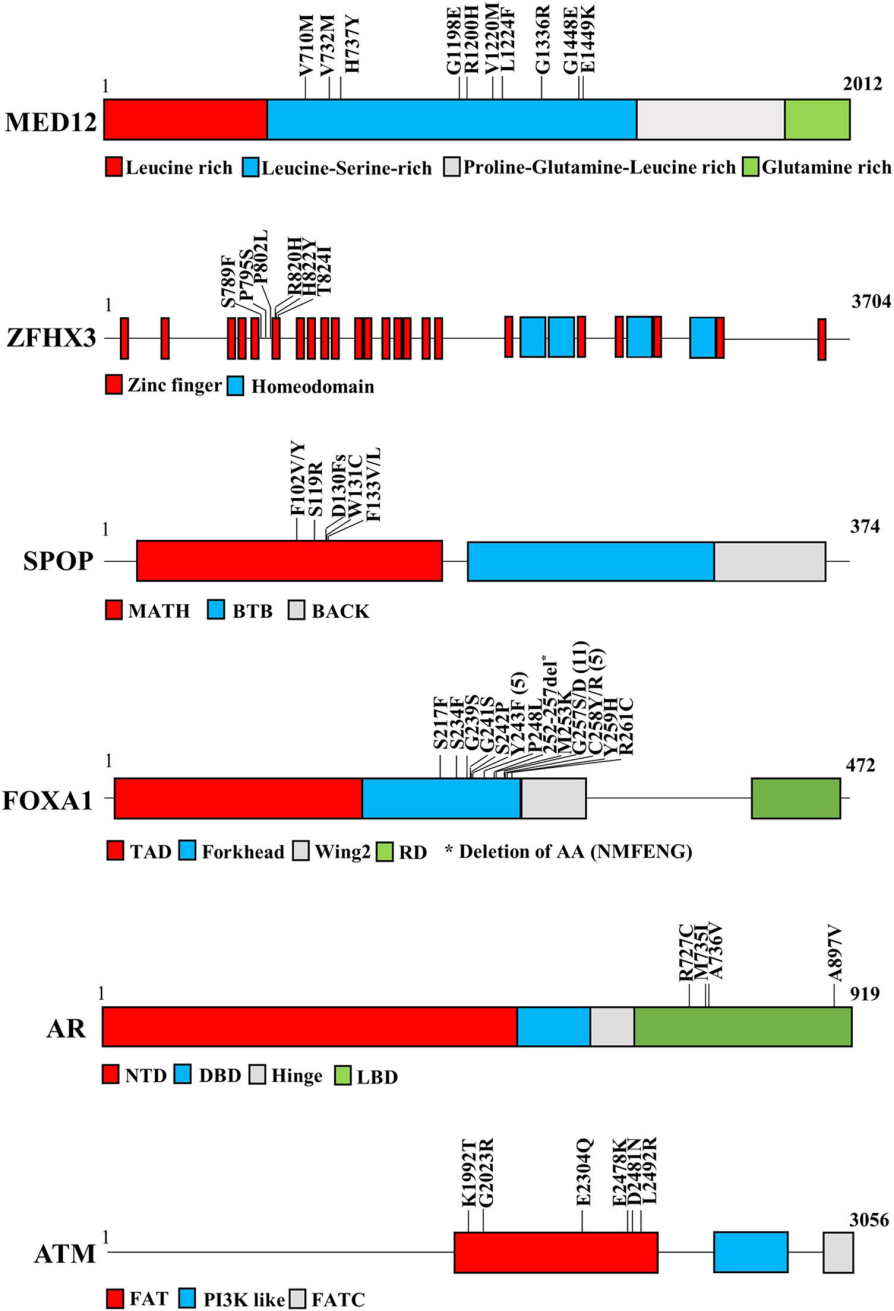
Hotspot regio.ns detected in *MED12, ZFHX3, SPOP, FOXA1, AR*, and *ATM*. Genes are represented as bars or boxes not to scale; protein domains and mutations are indicated.

We found hotspot mutations in different genes (Figure 3); in particular, the most of *MED12* variants (91%) lay in the leucine‐serine‐rich domain, where three of these were close together and the others widespread along this domain. Regarding *ZFHX3*, hotspot mutations were detected in the protein segment between the fifth and sixth zinc finger domain and about 24% of these variants hit few codons (amino acids 789–824).

Hotspot mutations in *SPOP* were also discovered; approximatively 87% of these variants lay very close together in the MATH domain. Interestingly, all the seven lesions found in the MATH domain were considered pathogenic, while the only one detected outside (E334D) was a polymorphism. Most of *FOXA1* mutations (62%) were clustered in a short protein segment (AA 217–261) of the Forkhead domain. In particular, all lesions were classified as pathogenic except the S217F and Y243F substitutions that were considered as uncertain significance.

We found that about 66% of mutations in *AR* were located in the ligand‐binding domain (LBD) and were characterized as pathogenic lesions. Three of these were close together, while the fourth was located at the end of LBD. Finally, we discovered several lesions (12%) localized in the FRAP‐ATM‐TRRAP (FAT) domain of *ATM*, where three of these variants lay very close together while the others were spread along this motif.

### 3.4 Linkage between gene mutation and disease outcome

**Figure 4:**
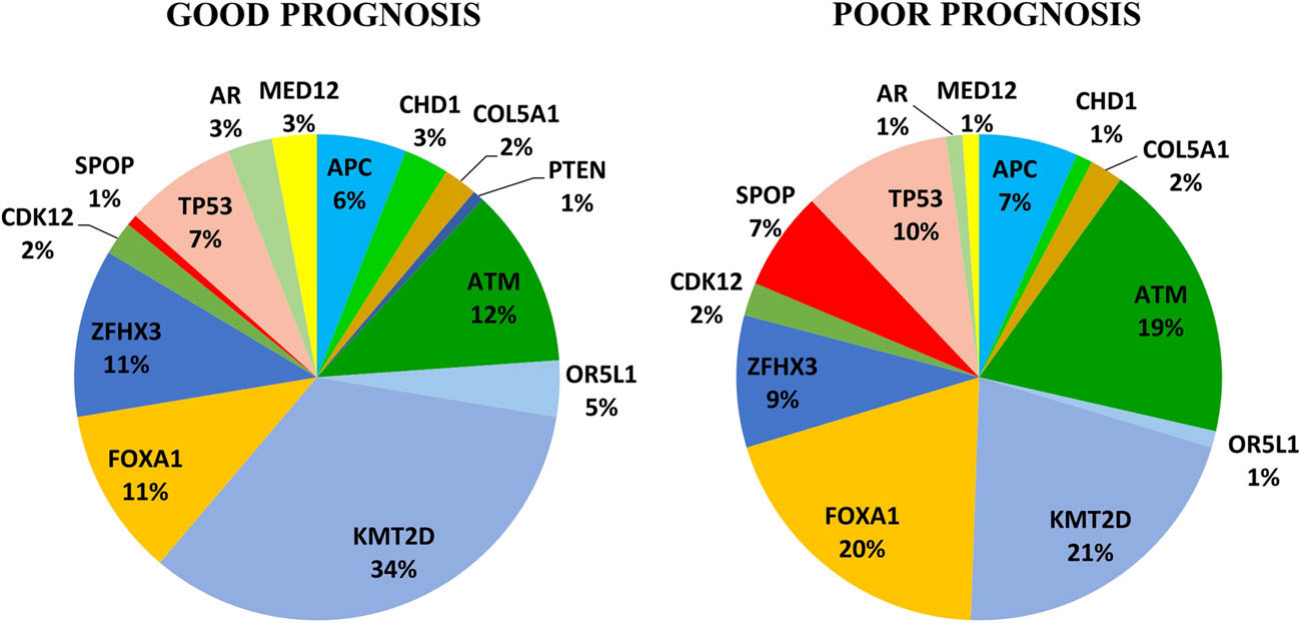
Pie chart showing the percentage of mutated genes in PCa patients with good (*n* = 20) or poor (*n* = 19) prognosis. Gene mutation percentage in PCa patients with poor or good prognosis is indicated. In more detail, patients with poor prognosis show increased mutation frequency in *FOXA1, SPOP, TP53*, and *ATM*. Conversely, the mutation frequency in *KMT2D, OR5L1, CHD1*, *AR*, and *MED12* is lower. The percentage of mutated genes for each group (good or poor) was calculated in comparison with all mutations (100%) detected in patients of that group. Mutations in *RB1* and *PIK3CA* were excluded because they are found only in patients without follow‐up data. PCa, prostate cancer

Mutations found in our cohort were matched with patient follow‐up data. As shown in Figure 4, the percentage of mutated genes between the group with good and poor prognosis was different. The mutation frequency of *MED12*, *AR*, *CHD1*, *OR5L1*, and *KTM2D* was lower in patients with poor prognosis. In particular, lesions found in *KMT2D* were much more common in the group of patient with good prognosis. Conversely, mutations detected in *FOXA1, SPOP, ATM*, and *TP53* were mainly found in patients with poor prognosis, while the mutation percentage of *APC*, *COL5A1*, *ZFHX3*, and *CDK12* was substantially unchanged. In more detail, different *FOXA1* variants laying in the forkhead domain were linked to biochemical recurrence as well as those found in *SPOP*. Moreover, the truncating lesions R805X and L2692X as well as the substitution R3008H in *ATM* were associated with poor prognosis. Similarly, lesions in *TP53* such as Y163H, T172Ifs, and R267P were associated with both higher Gleason score and tumor progression (Table 2).

**Table 2:** Clinical parameters of 48 prostate cancer patients.

Acronym	Age	Diagnosis	PSA value	Surgical resection	Outcome	Therapy	Mutated genes	Notes
B4536	62	Prostate adenocarcinoma Gs 6 (3 + 3)	0.41	2012	Biochemical recurrence	Rescue radiation therapy	*ATM, FOXA1*	None
B4972	66	Prostate adenocarcinoma Gs 6 (3 + 3)	0.02	2012	No metastasis	None	*KMT2D*	Very low mutation frequency
B6393	Deceased	Prostate adenocarcinoma Gs 6 (3 + 3)	Undetectable	2012	Deceased for oligodendroglioma no PCa metastasis	None	*ATM, APC*	Very low mutation frequency
B6998	70	Prostate adenocarcinoma Gs 6 (3 + 3) and colon adenocarcinoma	Undetectable	2012	Abdominal lymph node metastasis	Chemotherapy	*ZFHX3*	Very low mutation frequency
B3059	71	Prostate adenocarcinoma Gs 6 (3 + 3)	NA	2012	Biochemical recurrence	Rescue radiation therapy	*ATM, ZFHX3*	Very low mutation frequency
B1845	71	Prostate adenocarcinoma Gs 6 (3 + 3)	NA	2013	No follow up	Unavailable data	none	No pathological mutations were detected
B992	80	Prostate adenocarcinoma Gs 6 (3 + 3) and urothelial carcinoma	NA	2013	No follow up	Unavailable data	ATM, KMT2D, TP53	*ATM* germline
B2726	75	Prostate adenocarcinoma Gs 6 (3 + 3) and colon adenocarcinoma	0.01	2013	Biochemical recurrence	Rescue radiation therapy	*KMT2D, SPOP*	*SPOP* (hot spot)
B3082	77	Prostate adenocarcinoma Gs 6 (3 + 3)	NA	2013	Biochemical recurrence	Rescue radiation therapy	*FOXA1*	Very low mutation frequency
B2508	72	Prostate adenocarcinoma Gs 6 (3 + 3)	NA	2013	No follow up	Unavailable data	none	No pathological mutations were detected
B501	73	Prostate adenocarcinoma Gs 6 (3 + 3)	NA	2011	No follow up	Unavailable data	*CHD1, APC, OR5L1, ATM, KMT2D, RB1, FOXA1, ZHFX3, SPOP, AR, MED12*	Multiple gene mutations
B8935	72	Prostate adenocarcinoma Gs 6 (3 + 3)	Undetectable	2011	No metastasis	None	*MED12, FOXA1*	Multiple mutations of *FOXA1* gene
B1387	81	Prostate adenocarcinoma Gs 6 (3 + 3)	Undetectable	2011	No metastasis	None	*CHD1, COL5A1, KMT2D, ZFHX3, MED12, FOXA1*	Multiple gene mutations
B1753	68	Prostate adenocarcinoma Gs 6 (3 + 3)	Undetectable	2011	No metastasis	None	*ATM, KMT2D, ZFHX3, CDK12*	Very low mutation frequency
B1806	77	Prostate adenocarcinoma Gs 6 (3 + 3)	Undetectable	2011	Biochemical recurrence	Rescue radiation therapy	*ATM, KMT2D, FOXA1*	None
B8502	63	Prostate adenocarcinoma Gs 6 (3 + 3)	NA	2011	No metastasis	None	*APC, ATM, KTM2D, CDK12, FOXA1*	Multiple mutations of *KTM2D*
B6265	78	Prostate adenocarcinoma Gs 6 (3 + 3)	NA	2011	No follow up	Unavailable data	*KTM2D, FOXA1, ATM*	None
B7149	85	Prostate adenocarcinoma Gs 6 (3 + 3)	Undetectable	2011	No metastasis	None	none	No pathological mutations were detected
B7595	67	Prostate adenocarcinoma Gs 6 (3 + 3)	Undetectable	2011	No metastasis	None	*KMT2D*	Very low mutation frequency
B7487	78	Prostate adenocarcinoma Gs 6 (3 + 3)	Undetectable	2011	No metastasis	None	*FOXA1*	None
B7756	75	Prostate adenocarcinoma Gs 6 (3 + 3)	Undetectable	2011	No metastasis	None	none	No pathological mutations were detected
B7360	81	Prostate adenocarcinoma Gs 6 (3 + 3)	Undetectable	2011	No metastasis	None	*ATM, TP53, MED12*	None
B8752	78	Prostate adenocarcinoma Gs 7 (3 + 4)	Undetectable	2011	No metastasis	Rescue radiation therapy	*ATM*	*ATM* germline
B8456	63	Prostate adenocarcinoma Gs 7 (3 + 4)	NA	2011	No metastasis	None	none	No pathological mutations were detected
B778	80	Prostate adenocarcinoma Gs 7 (3 + 4)	NA	2011	Biochemical recurrence	Rescue radiation therapy	*ATM, FOXA1*	*KMT2D* germline
B8135	83	Prostate adenocarcinoma Gs 7 (3 + 4)	NA	2011	No metastasis	None	*PTEN, ATM*	*ATM* germline
B7970	81	Prostate adenocarcinoma Gs 7 (3 + 4)	Undetectable	2011	No metastasis	None	*CDK12, AR, FOXA1*	Very low mutation frequency
B8234	74	Prostate adenocarcinoma Gs 7 (3 + 4)	Undetectable	2011	Biochemical recurrence	Rescue radiation therapy	*KMT2D, MED12, FOXA1*	None
B6286	73	Prostate adenocarcinoma Gs 7 (3 + 4)	Undetectable	2011	Biochemical recurrence	Rescue radiation therapy	*ATM, SPOP, FOXA1*	*SPOP* (hot spot)
B6547	78	Prostate adenocarcinoma Gs 7 (3 + 4)	Undetectable	2011	Biochemical recurrence	Rescue radiation therapy	*CHD1, ZFHX3, TP53, SPOP, FOXA1*	*SPOP* (hot spot)
B6607	78	Prostate adenocarcinoma Gs 7 (3 + 4)	Undetectable	2011	Biochemical recurrence	Rescue radiation therapy	*APC*	Very low mutation frequency
B6055	69	Prostate adenocarcinoma Gs 7 (3 + 4)	NA	2011	No follow up (alive)	Unavailable data	*KMT2D, FOXA1*	None
B6395	79	Prostate adenocarcinoma Gs 7 (3 + 4)	Undetectable	2011	No metastasis	None	*KMT2D, FOXA1*	*KMT2D* germline
B6820	75	Prostate adenocarcinoma Gs 7 (3 + 4)	NA	2011	No follow up (alive)	Unavailable data	*PI3KCA, ZFHX3, PTEN*	None
B6224	72	Prostate adenocarcinoma Gs 8 (3 + 5), basocellular carcinoma and squamous cell carcinoma (skin)	NA	2011	Lymph node metastasis; total androgen blockade	LHRH analog	*APC, KMT2D, ATM, FOXA1*	None
B8118	77	Prostate adenocarcinoma Gs 8 (4 + 4)	NA	2011	Follow up until 2013	Postsurgical radiotherapy and hormone therapy	*MED12, FOXA1, RB1*	None
B8519	79	Prostate adenocarcinoma Gs 8 (4 + 4)	NA	2010	No follow up (alive)	Unavailable data	*COL5A1, ATM, KMT2D, FOXA1*	None
B5172	65	Prostate adenocarcinoma Gs 8 (3 + 5)	NA	2012	No metastasis	Postsurgical radiotherapy and hormone therapy	*ATM*	Very low mutation frequency
B1658	77	Prostate adenocarcinoma Gs 8 (3 + 5)and basocellular carcinoma	NA	2013	Biochemical recurrence	Rescue radiation therapy	*ATM, SPOP*	SPOP (hot spot)
B2325	71	Prostate adenocarcinoma Gs 8 (4 + 4)	NA	2013	Lymph node metastasis	Unavailable data	*ATM, SPOP, KTM2D, AR*	*SPOP* (hot spot)
B779	77	Prostate adenocarcinoma Gs 8 (5 + 3)	NA	2013	Bone metastasis; total androgen blockade	Vertebral radiotherapy	*ATM*	*ATM* germline
B2471	70	Prostate adenocarcinoma Gs 8 (5 + 3)	NA	2013	Biochemical recurrence	Rescue radiation therapy	*ATM, SPOP*	*ATM* germline, *SPOP* (hot spot)
B3064	77	Prostate adenocarcinoma Gs 8 (3 + 5) and squamous cell carcinoma (palate)	NA	2013	No metastasis	Postsurgical radiotherapy	*FOXA1*	none
B6007	61	Prostate adenocarcinoma Gs 8 (4 + 4) and b cell lymphoma cutaneous	NA	2013	No metastasis; total androgen blockade	Postsurgical radiotherapy	*APC, ATM, KMT2D*	Very low mutation frequency
B4441	Deceased	Prostate adenocarcinoma Gs 8 (4 + 4)	NA	No surgery	Bone and visceral metastases	Bone radiotherapy, hormone therapy, Cabazitaxel	*ATM, TP53*	*TP53* germline
B435	Deceased	Prostate adenocarcinoma Gs (5 + 4), urothelial carcinoma and squamous cell carcinoma (lung)	NA	2013	Lung cancer relapse	Chemotherapy for lung cancer	*KMT2D*	*KMT2D* germline
B2777	62	Prostate adenocarcinoma Gs 8 (4 + 5)	NA	2013	Lymph node metastasis	Hormone therapy, radiotherapy	*TP53*	None
B47	Deceased	Prostate adenocarcinoma Gs (4 + 5), squamous cell carcinoma (larynx) and acinar lung adenocarcinoma	NA	2014	Lymph node metastasis	Hormone therapy, radiotherapy	*TP53, CDK12*	*CDK12* germline

*Note*: Patient features, outcome, and putative causing‐disease mutated genes are indicated.

Abbreviations: NA, not available; PSA, prostate‐specific antigen.

### 3.5 Germline mutations and cancer familiarity

**Figure 5:**
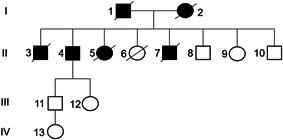
Pedigree of a case with the germline mutation R805X in *ATM*. Subjects 1 and 2 are deceased for gastric and lung cancer, respectively; Cases 3 and 7 are deceased for lung carcinoma and Subject 5 is dead for a hematological disease. The proband (Case 4) is alive and he suffered from prostate cancer, cholangiocarcinoma, melanoma, and two lung cancers.

We detected different germline variants with likely pathological significance and possible hereditary predisposing‐cancer syndrome in our PCa cohort. In particular, these germline mutations were observed in 10 patients (about 20%) and hit several genes including *ATM*, *KMT2D*, *TP53*, and *CDK12*. Many germline mutations were found in cases with metastasis and high Gleason score. In fact, of the 10 patients with germline variants, two had a Gleason score 9, three 8, four 7, and only one subject 6. The germline variants R3008H and R805X in *ATM* as well as the substitution P1275L in *CDK12* correlated with cancer familiarity. In particular, we found that the mother of the case carrying the R3008H substitution suffered for breast cancer, while the patient carrying the truncating mutation R805X showed a severe cancer familiarity. His father suffered for gastric carcinoma, while his mother was diagnosed with lung cancer. In addition, two brothers died for lung carcinoma and a sister was deceased for blood cancer (Figure 5). Finally, the mother of the case with the P1275L substitution in *CDK12* suffered for breast cancer. No hereditary cancer predisposition linked to the germline mutations K1992T, G2023R, and L2492R in *ATM* as well as R466C, R5229H, and S5357T in *KMT2D* were observed (Table 3).

**Table 3:** Patients carrying mutations linked to hereditary predisposing syndrome

Sample ID	Gene	Mutation	Frequency (%)	AA change	dbSNP	Mutation pathogenicity	Outcome	Familiarity	References
B8752	ATM	G6067A	48.5	G2023R	rs11212587	Uncertain	Alive (no metastasis)	None	Tsaousis et al. (2019)
B8135	ATM	T7475G	49.2	L2492R	rs56399857	Uncertain	Alive (no metastasis)	None	Tsaousis et al. (2019)
B2471	ATM	G9023A	49.8	R3008H	rs587781894	Pathogenic	Alive (biochemical recurrence)	Mother with breast cancer	Paglia et al. (2010)
B992	ATM	A5975C	45.9	K1992T	rs150757822	Uncertain	ND	ND	Tsaousis et al. (2019)
B779	ATM	C2413T	48.3	R805X	none	Pathogenic	Bone metastasis	Mother with lung cancer and father with gastric carcinoma	Karlsson et al. (2021)
B6395	KMT2D	G16070C	46.1	S5357T	none	Uncertain	Alive (no metastasis)	None	None
B778	KMT2D	C1396T	45.9	R466C	rs201512665	Uncertain	Alive (biochemical recurrence)	None	None
B435	KMT2D	G15686A	49.8	R5229H	rs201628357	Uncertain	Deceased (lung cancer)	None	None
B4441	TP53	G800C	56.1	R267P	rs587780075	Likely pathogenic	Deceased (bone metastasis)	ND	Giacomelli et al. (2018)
B47	CDK12	C3824T	47.5	P1275L	rs34070318	Uncertain	Deceased (lung cancer and laryngeal carcinoma)	Mother with breast cancer	Jiang et al. (2018); Pratz et al. (2016)

*Note*: Mutations, patient outcomes, and familiarity are included.

## 4 DISCUSSION

The most common alteration found in prostate cancer is the fusion between the androgen‐regulated *TMPRSS2* gene and *ERG* oncogene which occurs in approximately 50% of cases (Alvarez‐Cubero et al., 2017). Nevertheless, it has been reported that translocations involving the ETS family members alone are not sufficient to induce prostate neoplastic transformation and additional alterations such as PTEN and TP53 loss of function could affect the clinical subtype of PCa (Shtivelman et al., 2014). Moreover, the fusion TMPRSS2‐ERG was mainly found in early stage of disease (Yamoah et al., 2021). It is mutually exclusive with other alterations including *SPOP* and *CHD1* loss of function, indicating that TMPRSS2‐ERG negative prostate cancers progress by different tumorigenic processes or represent different cellular subtypes (Shtivelman et al., 2014; Yamoah et al., 2021; Zhu et al., 2021). Thus, the use of new powerful technologies in particular NGS could facilitate the discovery of new somatic and germline mutations improving prognosis and therapeutic response (Alvarez‐Cubero et al., 2017).

The analysis of gene variants in our prostate cancer cohort by NGS shows multiple mutations in different genes that may affect signaling pathways involved in prostate carcinogenesis. In particular, we have analyzed the impact of mutations on several biological processes linked to DNA instability and proliferative signals as well as germline variants associated with hereditary cancer syndrome.

### 4.1 DNA repair network

Many genes including *ATM*, *CDK12*, *SPOP*, and *CHD1* belonging to DNA repair machinery are mutated in PCa and their dysfunction causes genomic instability. Mutations in *ATM* including the FAT domain were found in PCa indicating that the dysfunction of this kinase may affect the fate of this tumor (Warner et al., 2021). However, these observations are debated since a recent study reports that *ATM* loss of function is not directly associated with worse outcomes, even if lesions of *ATM* increase the genomic instability (Neeb et al., 2021). We have detected several mutations of *ATM* lying in the FAT domain that does not correlate with poor prognosis in our cohort. They are already detected in breast cancer and chronic lymphocytic leukemia (Austen et al., 2007; Bernstein et al., 2010; Podralska et al., 2018) suggesting that these variants might affect cancer development. Outside the FAT domain, we have identified other mutations including the missense variant E365K, processed as uncertain significance, that is very frequent in our cohort, but does not correlate with cancer progression. Conversely, the truncating lesions R805X and L2692X as well as the variant R3008H, defined as pathogenic, are linked to poor prognosis. *ATM* mutations could alter the DNA damage response (DDR) machinery leading to genomic instability and acquisition of subsequent mutations that could affect prostate carcinogenesis. In different patients with *ATM* mutations, we have detected lesions in other genes including *ZFHX3, FOXA1* and *SPOP* that are frequently mutated in PCa patients. In particular, the dysfunction of SPOP, that is, another gene implicated in DNA repair is associated with cancer progression (García‐Flores et al., 2014; Ma et al., 2018). The analysis of *SPOP* variants shows that all pathogenic mutations are localized in a hotspot region within the MATH domain, which is responsible for substrate binding (Ma et al., 2018). Mutations of residues F102, S119, W131, and F133 are already observed in PCa (Barbieri et al., 2012; Boysen et al., 2015; Ma et al., 2018), while the lesion D130fs has never been detected before. The linkage between *SPOP* mutations and poor prognosis is not well defined, because some authors report that the impairment of *SPOP* function is associated with less adverse pathologic features and a favorable prognosis (Liu et al., 2018). Our observations indicate that all *SPOP* pathogenic lesions are associated with patients that have developed biochemical recurrence or lymph node metastasis, but they do not correlate with the most serious cases.

No linkage between *CHD1* and *CDK12* mutations and cancer progression has been observed in our cohort except for the germline variant P1275L in *CDK12* that will be discussed later.

### 4.2 AR signaling dysfunction

The alteration of androgen receptor‐regulated signaling may affect prostate cancer development and progression. In fact, *AR* point mutations range from 15% to 30% of patients with metastatic PCa (Fujita & Nonomura, 2019). In our cohort, we have detected the likely pathogenic mutations R727C, M735I, and A736V in *AR* that are localized in LBD domain. The substitution R727C was also found in patients with 46 XY disorders of sex development (DSD) (Ittiwut et al., 2017), while the other two are new variants. It was reported that the relevance of AR mutations in patients with advanced PCa remains unclear (Eisermann et al., 2013). In this study, we have not found *AR* mutations associated with poor prognosis. On the other hand, most *AR* lesions linked to worse outcomes are splice variants (AR‐Vs), which are constitutively activated by the truncation of the COOH‐terminal domain (Antonarakis et al., 2016).

Mutations of FOXA1, a protein that functions as a pioneer factor to facilitate AR transactivation and PCa growth (Zhao et al., 2014), are very frequent in our cohort. FOXA1 is a transcription factor that modulates AR‐driven transcription and mutations strictly affected residues of the Forkhead domain in PCa (Barbieri et al., 2012). Consistently, the most of *FOXA1* mutations detected in our cases lie in a hotspot region of the forkhead domain. M253K, C258Y/R, Y259H, and R261C substitutions were already described (Adams et al., 2019; Barbieri et al., 2012; Ritter et al., 2020), but the other lesions found in this domain are novel mutations. Variants of forkhead domain likely cause the alteration of protein function leading to cancer development and progression (Adams et al., 2019). Moreover, mutations in this region promote PCa progression regulating the expression of genes that mediate EMT and metastasis (Gao et al., 2019). Furthermore, it was observed that *FOXA1* mutations are associated with a worse clinical outcome (Shah & Brown, 2019). In our cases, most of the mutations found in forkhead domain of *FOXA1* are associated with biochemical recurrence.

### 4.3 Tumor suppressor proteins

Many tumors including prostate cancer rise, develop, and expand due to mutation in tumor suppressor genes including *KMT2D, PTEN*, *RB1*, *TP53*, and *ZFHX3*. *KTM2D* is the most mutated gene in our cohort. Eighty‐three mutations were detected in this gene suggesting that the dysfunction of this protein may affect prostate carcinogenesis. In fact, it is emerging that this gene is one of the most frequently mutated in a variety of tumors including PCa (Guo et al., 2013). Moreover, mutations in *KMT2D* are more frequent in metastatic than in primary tumors (Testa et al., 2019). In contrast to these observations, we report that mutations of *KTM2D* are prevalent in PCa patients with good outcome. On the other hand, the most of *KMT2D* mutations found in our cases have a low frequency or are classified as benign except the somatic stop gain E568X that is associated with biochemical recurrence. The germline variants R466C, R5229H, and S5357T will be discussed later.

Most of *PTEN* and *RB1* mutations found in our cohort are variants with uncertain significance and do not correlate with tumor progression. We have also identified the pathogenic truncating lesion C211Lfs in *PTEN*, but unfortunately, no follow‐up data for the case carrying this variant are available.

Many mutations in *ZFHX3*, a tumor suppressor gene frequently mutated in prostate cancer (Sun et al., 2005, 2015), were identified. These are mainly clustered in a region lying between the fifth and sixth zinc‐finger domain. It has been reported that the inactivation of *ZFHX3* may correlate with tumor aggressiveness, especially in subjects with the deletion of chromosome 16q that contains this gene (Sun et al., 2005). No linkage between *ZFHX3* mutations and poor prognosis we have observed, probably because several variants are considered benign or with uncertain significance while those characterized as likely pathogenic have low frequency and could be irrelevant for disease progression. On the contrary, different pathogenic mutations in *TP53* correlate with worse outcomes in our PCa cases; in particular, the mutations Y163H, T172Ifs, and R267P were detected in patients with metastasis. The mutation V274A also considered pathogenic is not linked to cancer progression, however, it was predominantly found in breast cancer (Végran et al., 2013). Lesions in *TP53* are associated with more aggressive disease not only in PCa but also in many other solid tumors (Mateo et al., 2020; Vodicka et al., 2021) and our data support these observations.

### 4.4 Cell growth and invasion

We have analyzed mutations in genes associated with cell proliferation and motility such as *COL5A1*, *PIK3CA*, *APC*, and *MED12*. Mutations found in *PIK3CA*, *COL5A1*, and *APC* have not a significant impact on patient outcomes in our cohort. Regarding *MED12*, it was reported that mutations in this gene are frequent in PCa (Barbieri et al., 2012). We have detected variants of *MED12* in 7 of 48 patients (14.5%). All pathogenic mutations detected in *MED12* lie in the leucine‐serin‐rich domain except the variant A157T, suggesting that this protein region may be involved in the tumorigenesis of PCa. Actually, this domain is strongly conserved and mutations located inside this region are associated with prostate tumor (Barbieri et al., 2012; Kämpjärvi et al., 2016). Interestingly, some studies report that the missense mutation L1224F is a recurrent variant in prostate cancer (Barbieri et al., 2012), while others did not observe this lesion in any of their cases (Stoehr et al., 2013). We have found this mutation solely in one subject with a low tumor stage and without metastasis. Moreover, *MED12* mutations found in our cohort do not correlate with cancer progression in most of cases, suggesting that MED12 dysfunction could not be associated with tumor metastasis.

### 4.5 Germline mutations and cancer familiarity

We have searched germline mutations that could be associated with inherited cancer. Ten variants in heterozygous form also expressed in normal tissue were detected in *ATM*, *KMT2D*, *TP53*, and *CDK12*. Germline mutations of *ATM* such as K1992T, G2023R, and L2492R have uncertain significance (Tsaousis et al., 2019); therefore, their role in hereditary cancer is not well defined. We have observed that cases carrying G2023R and L2492R mutations have neither metastasis nor cancer familiarity, while no information on clinical outcome for the patient with the K1992T variant is available. On the contrary, the subject carrying the germline mutation R3008H has developed biochemical recurrence and his mother suffered from breast cancer. Accordingly, this lesion has been already associated with hereditary breast cancer (Paglia et al., 2010), but in PCa it was never found before. Interestingly, one case carrying the truncating variant R805X in *ATM* has suffered for five different cancers and shows a severe cancer familiarity. In particular, mother and father are deceased for lung and gastric cancer, respectively. Furthermore, four siblings are deceased; two brothers with lung cancer, one sister for leukemia, and the second for a disease not linked to cancer (pedigree of Figure 5). The proband is alive and, in addition to prostate cancer, two lung tumors, one cholangiocarcinoma, and one melanoma were diagnosed. Currently, the truncating variant R805X has been described only in breast cancer, however truncating mutations in *ATM* such as stop gain or frameshift were also found in familial PCa (Karlsson et al., 2021). In addition, germline mutations of *ATM* are associated with gastric cancer as well as lung carcinoma (Huang et al., 2015; Parry et al., 2017). Taken together, these observations suggest that the lesion R805X could be associated with a high risk to develop tumors; moreover, *ATM* pathogenic germline lesions could be considered possible markers for familial cancer.

We have found germline mutations also in *KMT2D*; the variants R466C, R5259H, and S5357T are classified as uncertain significance and none of these is associated with familial cancer. However, patients carrying the R466C and R5229H substitutions have developed biochemical recurrence and lung cancer, respectively. Consistently, it is known that *KMT2D* is among the most highly inactivated epigenetic modifiers in lung cancer (Alam et al., 2020). Interestingly, in a subject with advanced PCa and bone metastasis, we have detected the germline mutation R267P in *TP53*. This variant causes the dysfunction of TP53 protein and was already detected in both liver and lung carcinoma (Giacomelli et al., 2018). Unfortunately, this patient is deceased and information about hereditary cancer predisposition is no longer available. Finally, we identified the germline mutation P1275L of *CDK12* in a case deceased for multiple cancers. In addition to PCa, this patient has suffered from lung carcinoma and laryngeal cancer; moreover, his mother is deceased of breast cancer. Importantly, in this patient, the somatic mutation Y163H in *TP53* that is associated with lung cancer was also detected (Vega et al., 1997). The germline variant P1275L was observed in myeloproliferative neoplasms and in EGFR‐mutated tumors (Jiang et al., 2018; Pratz et al., 2016), but its role in both prostate and breast cancer should be further investigated.

## 5 CONCLUSIONS

NGS analysis performed in 48 normal and corresponding prostate cancer tissues has allowed the detection of several lesions in *TP53*, *ATM*, *FOXA1*, and *SPOP* associated with cancer progression. Moreover, we described first‐time hotspot mutations in *ZFHX3* and novel mutations in the hotspot region of *FOXA1*. Furthermore, this study has led to the identification of different germline mutations, some of which in cases with familial cancer were found.

Our data indicate that mutations detected mainly in *ATM* and *TP53* could be used as biomarkers for poor prognosis in prostate cancer. Moreover, mutations altering pathways involved in prostate carcinogenesis including FOXA1‐, SPOP‐ and ATM‐regulated signals could be useful to discover new therapeutic targets for the treatment of metastatic PCa.

## AUTHOR CONTRIBUTIONS

Gianluca Aguiari and Alessandra Mangolini designed the project. Christian Rocca, Carmelo Ippolito, Lucio Dell' Atti, Giovanni Lanza, and Roberta Gafà collected the samples and managed patient follow up. Alessandra Mangolini and Nicoletta Bianchi performed the experiments. Alessandra Mangolini, Cristian Bassi, and Gianluca Aguiari analyzed the data. Paolo Pinton, Massimo Negrini, and Gianluca Aguiari discussed the experiments. Gianluca Aguiari wrote the manuscript.

## CONFLICTS OF INTEREST

The authors declare no conflicts of interest.

## Supporting information

Supporting information.

## Data Availability

The data that supports the findings of this study are available in the supplementary material of this article.
